# Expression of Stretch-Activated Two-Pore Potassium Channels in Human Myometrium in Pregnancy and Labor

**DOI:** 10.1371/journal.pone.0012372

**Published:** 2010-08-25

**Authors:** Iain L. O. Buxton, Cherie A. Singer, Jennifer N. Tichenor

**Affiliations:** Department of Pharmacology and Department of Obstetrics and Gynecology, School of Medicine, University of Nevada, Reno, Nevada, United States of America; Institute of Zoology, Chinese Academy of Sciences, China

## Abstract

**Background:**

We tested the hypothesis that the stretch-activated, four-transmembrane domain, two pore potassium channels (K2P), TREK-1 and TRAAK are gestationally-regulated in human myometrium and contribute to uterine relaxation during pregnancy until labor.

**Methodology:**

We determined the gene and protein expression of K2P channels in non-pregnant, pregnant term and preterm laboring myometrium. We employed both molecular biological and functional studies of K2P channels in myometrial samples taken from women undergoing cesarean delivery of a fetus.

**Principal Findings:**

TREK-1, but not TREK-2, channels are expressed in human myometrium and significantly up-regulated during pregnancy. Down-regulation of TREK-1 message was seen by Q-PCR in laboring tissues consistent with a role for TREK-1 in maintaining uterine quiescence prior to labor. The TRAAK channel was unregulated in the same women. Blockade of stretch-activated channels with a channel non-specific tarantula toxin (GsMTx-4) or the more specific TREK-1 antagonist L-methionine ethyl ester altered contractile frequency in a dose-dependent manner in pregnant myometrium. Arachidonic acid treatment lowered contractile tension an effect blocked by fluphenazine. Functional studies are consistent with a role for TREK-1 in uterine quiescence.

**Conclusions:**

We provide evidence supporting a role for TREK-1 in contributing to uterine quiescence during gestation and hypothesize that dysregulation of this mechanism may underlie certain cases of spontaneous pre-term birth.

## Introduction

Premature birth is now the leading cause of newborn death worldwide [1] and comparable to the number of deaths from HIV/AIDS [2]. It accounts for 12% of all live births in the United States [3], 75% of all perinatal complications, the leading cause of fetal death [4] and is inexplicably more likely to occur in African American mothers [5]. Hospital charges for premature infants in the United States are 10 times that of a typical newborn [6] and the costs to society in the U.S.A. are estimated by the Institute of Medicine at $62 billion annually [7]. Despite improvements in prenatal, perinatal and neonatal care, the incidence of premature birth persists and is increasing [4]. To date, there is no effective means of treatment to prevent preterm delivery [8]. Indeed, until the development of the oxytocin receptor antagonist atociban (Tractocile®, Ferring Pharmaceuticals), no treatment was developed specifically based on myometrial pharmacology, the introduction of the β2 adrenergic agonist ritodrine notwithstanding. Administration of 17-hydroxyprogesterone caproate (17P) has held promise in the early clinical trial setting but it does not improve outcomes in twin pregnancies [9], nor does it appear to be generally useful in the clinical setting [10] although certain groups of patients may benefit [11]. Since the trigger(s) for preterm labor are not exclusively the actions of oxytocin and the enhanced contractility of laboring human myometrium is not all blocked by atosiban [12], the drug has not offered a major therapeutic advance. Treatment of mothers with so-called tocolytics (MgSO_4_, terbutaline, nifedipine) is ineffective beyond 48 hours and not without consequence [13], [14]. Indeed, the therapeutic focus of tocolytic use is providing time to employ steroid to mature the fetal lung rather than preventing delivery until term. It is imperative that we understand the unique physiological mechanisms underlying pregnancy and parturition at the biochemical and molecular level in order to discover new approaches to the prevention of preterm labor.

We previously reported that calcium-activated potassium channels (K_Ca_) are differentially regulated during gestation and suggested these channels as putative nitrosylation targets [15], [16]. Furthermore, recent studies from gastrointestinal (GI) smooth muscle have suggested that part of the hyperpolarizing effects of NO may be mediated by stretch-activated potassium (K2P) channels [17]. These mechanosensitive potassium channels are thought to help maintain relaxation of myocytes in visceral hollow organs by hyperpolarizing the membrane and have been found to regulate responses to nitrergic stimulation [18]. Although claimed to be absent in murine myometrium [18], we have described the expression of the potassium channels, subfamily K, member 4 (KCNK4) a two-pore potassium channel (K_2P_4.1) known as TRAAK (TWIK-related arachidonic acid-stimulated K^+^ channel) and member 2 (KCNK2) a two-pore potassium channel (K_2P_2.1) known as TREK-1 (TWIK-related K^+^ channel) in human myometrium [19] as did Bai et al. in the same year [20]. Given these observations, we became interested in the regulation of expression and signaling of these K2P channels in human pregnancy myometrium in term and preterm labor. Since the uterus undergoes unprecedented expansion and stretch during gestation, we suggest that the stretch-activated K2P channels are involved in the maintenance of uterine quiescence prior to the onset of labor.

Stretch-activated K2P channels make up a unique subset of K^+^ channels that are mechano-sensitive and belong to a larger family of channels characterized by four transmembrane segments (TMS) and two pore (2P) domain regions. Unlike other members of the 4TMS/2P channel family TREK-1, TREK-2, and TRAAK belong to the TRAAK-family subset of K2P channels (KCNK2, KCNK10 and KCNK4) that are activated by arachidonic acid and increased membrane tension [21]–[23], both of which play a role during parturition [24], [25]. These channels which are thought to form mature channels as homo- and or heterodimers, are also known as leak or background K^+^ channels and play an essential role in setting the resting membrane potential of myocytes [23], [26]. Examination of the literature reveals conflicting evidence for the expression of these K2P channels in uterine muscle [18], [20], [27], [28]. Therefore, the expression and differential regulation of TRAAK-family channels during pregnancy and labor in human myometrium is of much interest, especially as they, or their regulation may represent potential therapeutic targets in pre-term labor.

Here we report that TRAAK-family members TREK-1 and TRAAK, but not TREK-2, are expressed in human pregnancy myometrium and that TREK-1 is differentially regulated during pregnancy. We provide evidence that inhibition of myometrial K2P channels (*i.e.,* TREK-1) increases uterine excitability while activation of these channels lowers it. This work suggests that TRAAK-family channels, especially TREK-1, may be important in regulating uterine relaxation during pregnancy and hints at the possible dysregulation of this mechanism in pre-term births.

## Methods

### Ethics Statement

The research presented here was reviewed and approved in writing by the University of Nevada Biomedical Review Committee (IRB) for the protection of human subjects in research.

### Tissue Collection

With informed consent obtained in writing, samples of non-pregnant and pregnant (laboring and non-laboring) uterine tissue were obtained either *via* hysterectomy in pre-menopausal women ≤43 y undergoing hysterectomy when no uterine pathology is present, or elective cesarean section. Samples of non-pregnant uterine tissue were taken from the mid body following inspection by the pathologist, while samples from pregnant women were taken from the upper portion of the transverse uterine incision. Women were selected at random without inclusion criteria other than a clinical decision to deliver a pregnancy by Caesarian section (Table 1). Exclusion criteria were age less than 18 years, multiple pregnancy, known illicit drug use, or HIV or hepatitis C infection. Within 20 min of their removal, fresh tissue samples were transported to the laboratory in cold physiological buffer containing (in mM): NaCl (120), KCl (5), KH_2_PO_4_ (0.587), Na_2_HPO_4_ (0.589), MgCl_2_ (2.5), Dextrose (20), CaCl_2_ (2.5), Tris (25), and NaHCO_3_ (5), adjusted to pH 7.4. Tissues collected for molecular biological studies were preserved and transported in RNAlater (Ambion, Austin, TX). Tissue collection was approved through the University of Nevada, Biomedical Institutional Review Board (IRB).

**Table 1:** Patient characteristics-pre-gravid tissues.

		Ethnicity			Diagnosis
	Age (y)	White	Latino	A. Am.	
Hysterectomy	(38–43)	4	8	4	Elective (all without disease)
**Patient Characteristics-Gravid Tissues**					
Term (38–41 wks.)	(22–38)	9	13	5	Elective C-Section (all)
Term in Labor (39–40 wks.)	(26–32)	4	7	2	Elective (8) Breech (3), Placenta Previa (2)
Preterm in Labor (26–34 wks.)	(24–30)	2	0	3	PROM (2), Cervical Dilation >7 cm (3)

Uterine smooth muscle (myometrium) was first dissected from human uterine tissue samples and then either flash frozen in liquid nitrogen and stored at −80°C for later analysis or immediately utilized in contractile studies.

### Semi-quantitative PCR

Total RNA was extracted from 50 mg (wet wt.) of myometrium in TRIzol (Invitrogen, Carlsbad, CA) according to the manufacturer's protocol and resuspended in 30 µl nuclease-free H_2_O. DNA contamination was removed by treatment at 37°C with 10 U RNAse-free DNAse I (Promega, Madison, WI). DNAse was inactivated by adding 25 mM EDTA with heating at 55°C for 10 min. cDNA was synthesized from 1 µg of total RNA using 250 ng random primers (Invitrogen), 0.125 mM each dNTPs, 10 mM DTT and 200 U Superscript II reverse transcriptase (Invitrogen).

Gene specific primers for human TRAAK, TREK-1, and TREK-2 (Table 2) were designed from areas of high homology between respective channel sequences from various published sequences using Integrated DNA Technologies Primer Quest software (Coralville, IA). Basic local alignment search tool (BLAST) searches were performed to confirm that primer sequences had no homology with any other known gene products. β-actin primers were designed to amplify both genomic (750 bp) as well as non-genomic products (500 bp) to control for genomic DNA contamination, while non-template controls ensured the integrity of the PCR reaction. Amplification was performed with the Quantum RNA 18S Internal Standards according to the manufacturer's protocol (Ambion). An optimized ratio (1∶20) of 18S rRNA primers was added to the reaction as endogenous standard along with competimer to modulate 18S amplification without affecting the gene-specific PCR targets. PCR amplification within the linear range was carried out in a thermocycler under the following conditions: 95°C for 10 min as an initial melt, followed by 40 cycles of 95°C for 45 sec, annealing between 58°C to 60°C for 45 sec, and extension at 72°C for 45 sec; followed by a final extension of 72°C for 5 min. We determined the linear range of our PCR reactions by increasing cycle number and resolving bands by electrophoresis. The products were visualized by ethidium bromide staining and quantitated by computer. Cycle number was plotted against the signal obtained in order to identify the exponential (linear) range and the plateau phase. When cycle number is plotted against the log of the signal, a straight line was obtained. Human brain served as a positive control for channel expression. PCR products were imaged with UV light on a gel-documentation apparatus from Alpha-Innotech (San Leandro, CA). K2P channel bands were quantified by densitometry and expressed relative to bands of 18S rRNA controls which were amplified in parallel from each sample. Nucleotide sequencing was performed by the University of Nevada Genomics Center.

**Table 2:** PCR primers.

Gene	GenBank Accession No.	Semi-Quantitative PCR primer sequence	Product size (bp)
TRAAK	AF247042	F: 5′-TCTCAAGGGCTTCGTTTCTGCTCT -′3R: 5′-ATTGATGCAGGCTTTGAGGCACAG -′3	240
TREK-1	NM_001017424	F: 5′-TGGCTGTGTACTCTTTGTGGCTCT -′3R: 5′-ACTCAGTCGCCTCCTGGTTTCTTT -′3	349
TREK-2	NM_021161	F: 5′-TTGTTGGCCTTGCCTACTTTGCAG -′3R: 5′-ACACACACACACACACACACAACG -′3	756

Gene	GenBank Accession No.	Quantitative PCR primer/probe sequence	Product size (bp)
TREK-1 (SybergreenQPCR)	AF129399	F: GGA ATT CCC CTC TTT GGT TTT CR: CAC TTT GGC AAT TCC TTT TCC A	82
18S (Sybergreen QPCR)	X03205.1	F: CAC GGC CGG TAC AGT GAA AR: AGA GGA GCG AGC GAC CAA	72
TRAAK (TaqmanQPCR)	NM_033310.2	TaqMan Gene Expression Assay Hs99999901_s1, Applied Biosystems	187
18S (TaqmanQPCR)	X03205.1	TaqManGene Expression Assay Hs00213267_m1, Applied Biosystems	101

### Quantitative Real Time PCR

Human myometrial tissues were homogenized in TRIzol reagent, total RNA was isolated, and cDNA was synthesized as described above from 25 mg (wet wt.) of myometrium and diluted 1∶5. TREK-1 QPCR was carried out using SYBR I green dye and TRAAK QPCR was carried out using Taqman gene expression assays (Table 2; Applied Biosystems, Foster City, CA), both using an ABI Prism 7000 sequence detection system.

Each SYBR green reaction (25 µl total) contained 2 µl cDNA for 18S, 10 µl cDNA for TREK-1, 12.5 µl SYBR Green PCR 2X Master Mix, and 400 nM forward and reverse primers. Samples were heated to 50°C for 2 min, melted at 95°C for 10 min, and then cycled 45 times at 95°C for 15 sec, followed by annealing and extension at 58°C for 1 min. A single final dissociation step included 95°C for 15 sec, 58°C for 20 sec, and 95°C for 15 sec. Amplification of the message was monitored by measuring the increase in fluorescence caused by SYBER I green binding to double-stranded DNA, resulting in an amplification plot of fluorescence *vs.* cycle number.

Each Taqman reaction (25 µl total) contained 2 µl cDNA for 18S, 8 µl cDNA for TRAAK, 12.5 µl Taqman Universal PCR 2X Master Mix, 900 nM forward and reverse primers, and 250 nM probe. Samples were heated to 50°C for 2 min, melted at 95°C for 10 min, and then cycled 45 times at 95°C for 15 sec and 60°C for 1 min. Amplification was monitored by measuring the increase in fluorescence caused by the 5′ to 3′ nucleolytic activity of the Amplitaq Gold enzyme cleaving the fluorescently-labeled probe, resulting in an amplification plot of fluorescence *vs.* cycle number.

For both methods, standard curves were generated for each target gene using serial dilutions of cDNA. The amount of specific target genes in unknown samples was calculated by measuring the cycle threshold (Ct) values and extrapolating starting copy numbers from standard curves. All samples were tested in triplicate and normalized to 18S rRNA amplified from respective samples to control for variations in sample quality. Non-template controls using water in place of cDNA were included in all QPCR plates to ensure the integrity of reaction components.

### Western Blotting

Flash frozen myometrial samples were homogenized and sonicated in buffer consisting of 1% (v/v) Triton X-100, 150 mM NaCl, 10 mM NaH_2_PO_4_, 5 mM EDTA, and 1X Halt™ Protease Inhibitor Cocktail (Pierce, Rockford, IL). This cell lysate was then centrifuged at 14,000× *g* at 4°C for 30 min. The supernatant from each sample was then tested for protein concentration *via* Lowry assay (Bio-Rad, Hercules, CA) using bovine serum albumin (BSA) as a standard.

Protein from lysate supernatants (30–40 µg) was boiled for 5 min in 1X denaturing sample loading buffer containing: 0.06 M Tris-HCL (pH 6.8), 10% glycerol (v/v), 2% SDS (w/v), 0.03% bromophenol blue (w/v), and 5% β-mercaptoethanol (v/v). Proteins were separated by electrophoresis in 10% polyacrylamide gels (Bio-Rad, Hercules, CA) and transferred to a nitrocellulose membrane. Membranes were blocked at 4°C overnight in a 1∶1 solution of Odyssey™ blocking buffer (Licor Biosciences, Lincoln, NE) and phosphate buffered saline [PBS; 137 mM NaCl, 2.7 mM KCl, 0.9 mM KH_2_PO_4_, 6.4 mM Na_2_HPO_4_, adjusted to pH 7.4]. The membranes were then labeled for either human TREK-1 (1∶1000 rabbit IgG; Santa Cruz Biotechnology, CA) or TRAAK (1∶1000 goat IgG; Santa Cruz Biotechnology). Respective secondary antibodies conjugated to either infrared 680 or infrared 800 fluorescent dye (1∶100,000; Invitrogen or Rockland Immunochemicals, Philadelphia, PA) were used for detection. Antibody incubations were carried out in 1∶1 Odyssey™ blocking buffer (Licor Biosciences) and PBS with 0.1% Tween-20 (v/v) at 4°C. Bands were visualized using an infrared imaging system (LI-COR Biosciences V2.04, NE) using both the 700 nm and 800 nm channels. Relative protein levels were quantified *via* densitometry and normalized to GAPDH (1∶1500 mouse IgG; Santa Cruz Biotechnology) for each individual sample.

Human brain was utilized as a positive for both TREK-1 and TRAAK protein expression. Cos-7 cells over-expressing TREK-1 were employed as an additional positive control for TREK-1 protein detection [29], as well as a negative cross reactivity control for TRAAK protein detection. Cos-7 cells were maintained in Dulbecco's Modified Eagle Media (DMEM) supplemented with 10% fetal bovine serum (FBS). TREK-1 Cos cell lysate supernatant was obtained and probed as with human myometrial samples.

### Contractile Studies

Fresh non-laboring and laboring pregnant human uterine smooth muscle was dissected in Krebs buffer without Ca^+2^ so that only myometrium was present. Myometrium was cut into thin strips (1×5 mm), mounted in organ baths (Radnoti, Monrovia, CA), and attached to isometric force transducers (Kent Scientific, Litchfield, CT) by metal clips. Once mounted, tissues were bathed in Krebs buffer with Ca^+2^ [(in mM): NaCl (118), KCl (4.75), CaCl_2_ (2.5), KH_2_PO_4_ (1.2), NaHCO_3_ (25), MgSO_4_ (1.2), dextrose (20), adjusted to pH 7.4] and maintained at 37°C, aerated with 95% O_2_/5% CO_2_, and loaded with initial tensions of 1.8 g force as we have previously described [19], [30]. During the course of a 1 hr equilibration period, all tissues were routinely challenged with oxytocin (OT, 100 nM) followed by washout after which, tissues became spontaneously active. Stretch-activated channel toxin, GsMTx-4 (Peptides, International, KY) was added to baths at concentrations ranging from 0.9 µM to 3.6 µM, while L-Methionine ethyl ester (L-Mee) was tested between the range of 30 µM to 10 mM and fluphenazine was employed at 100 µM. Iberiotoxin (100 nM) was applied to all tissues to inhibit large-conductance calcium activated K^+^ channels. Transducer voltages were amplified and converted to digital signals by an analogue-to-digital board mounted within a computer system running the DASYLab data acquisition system (V10.0; IOtech, OH). Tissue contraction was compared and normalized to its own basal relaxed (control) state; contractility in the presence of varying levels of GsMTx-4 or L-Mee was quantified by comparing frequency of contraction or tension generated (grams) over time. GsMTx-4 has been reported to be a specific stretch-activated channel inhibitor [31], [32], while L-methionine and fluphenazine show reasonable selectivity as TREK channel antagonists [17], [33], [34].

### Statistical Analyses

All graphs were prepared using Prism Graphing Software (V5.01; GraphPad Software, CA) and power calculations and statistical analyses were carried out using Statmate and InStat Statistical Software (GraphPad Software, CA), with *p*≤0.05 considered significant. Significance was determined using either non-parametric analysis of variance (Kruskal–Wallis) with Dunn's multiple comparisons post-test or nonparametric t-test (Mann–Whitney). Data points and error bars represent means ± S.E.M. *, *p*≤0.05; **, *p*≤0.01; ***, *p*≤0.001 (between groups). For protein, transcript, and contractile experiments, the number of different patients and their respective myometrial tissue is denoted by ‘*n*’.

## Results

### TRAAK Gene and Protein Expression in Human Myometrium

**Figure 1:**
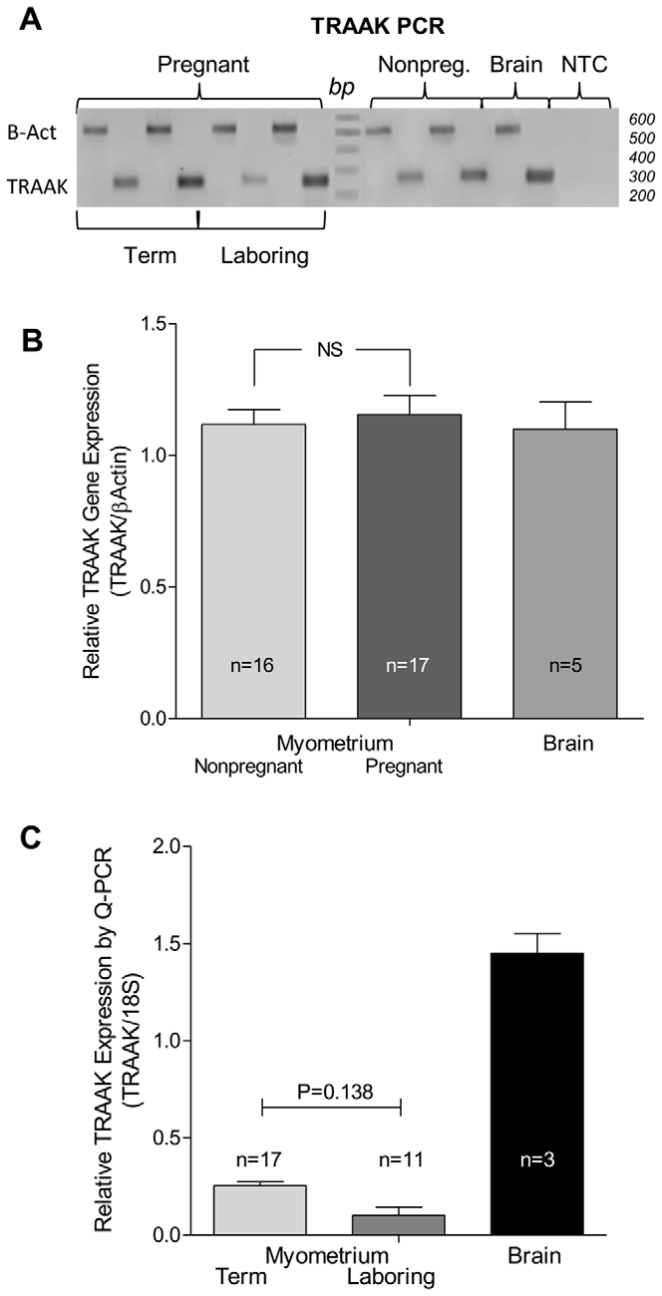
TRAAK K2P channel expression in human myometrium. (A) PCR studies were carried out on both non-pregnant and pregnant tissues and human brain. (NTC, non template control). (B) Human brain served as a control and β-actin (500 bp) was used to adjust TRAAK gene expression (240 bp) relative to control gene expression. PCR transcripts at 240 base pairs were sequenced and matched known TRAAK sequence. (C) Expression of TRAAK in term and term laboring samples and in brain as a comparative control was determined by Q-PRC with the 18S ribosomal gene as an expression control.

Relative myometrial TRAAK mRNA expression was determined by PCR and normalized to β-actin expression (Fig. 1A). TRAAK primers (Table 2) gave a 240 base pair transcript found to be expressed in both pregnant and non-pregnant human myometrial smooth muscle (Fig. 1B) at similar levels. Human brain was included as a positive control for TRAAK expression. The TRAAK PCR product was subsequently purified and sequenced by the Nevada Genomics Center and compared with known sequence using BLAST (National Center for Biotechnology Information) to further confirm its identity (98% identity). We determined that both pregnant and non-pregnant human myometrial smooth muscles express TRAAK with comparable abundance using this technique suggesting that there is no up-regulation of TRAAK channel gene expression during gestation (Fig. 1B).

In order to determine whether or not TRAAK expression in pregnant myometrium might be regulated at the time of labor, we used quantitative PCR (Fig. 1C). Myometrial gene expression was determined as normalized for expression of 18S ribosomal RNA that we have previously shown to be stable in non-pregnant versus pregnant myometrium [15]. A difference in TRAAK gene expression in laboring tissues taken from 13 women at term (39–40 weeks gestation) versus non-laboring tissues taken from 27 women at term (38–41 weeks gestation) was not significant. Power analysis confirmed that we would measure a difference if one existed at this level with 99% power to detect a significant difference at *p* = 0.05.

**Figure 2:**
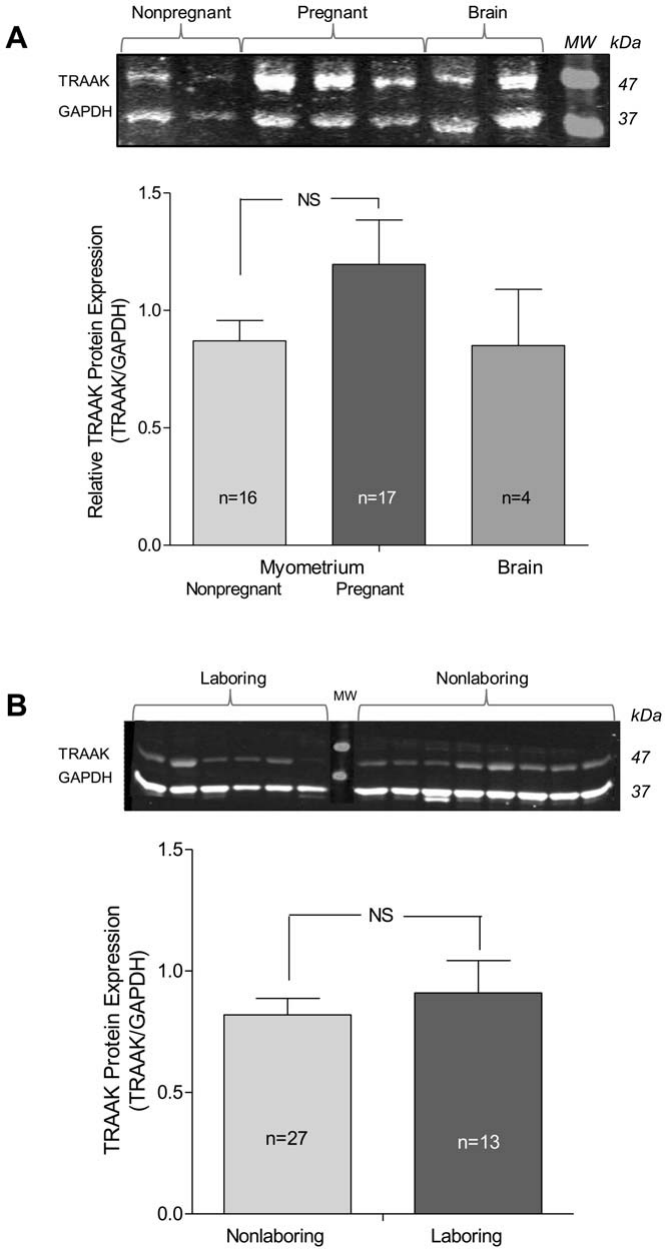
TRAAK protein expression in human myometrium. (A) Western blots were performed using a TRAAK-specific antibody (Santa Cruz) and stained using infrared fluorescence secondary from Licor Biosciences®. Individual lanes represent different patient samples and are shown to illustrate representative variation. GAPDH was employed as an expression control using simultaneous imaging. TRAAK expression is not increased by pregnancy compared to non-pregnant samples, nor was there any effect of labor (B). Representative Western blots are shown along with average data. Experiments were repeated three times on the same sample set and the results averaged for each sample. Data are Mean ± SEM compared by one-way analysis of variance.

TRAAK protein expression in myometrial samples was determined using Western blot (Fig 2A). Control experiments using TREK-1 over expressing COS cells demonstrated that the TRAAK antibody (*Santa Cruz, CA*) was specific for TRAAK expression (*data not shown*). TRAAK antibody revealed a single band at 47 kDa (under reducing conditions) and immunoblots were developed for GAPDH as a loading control and to normalize TRAAK expression (Fig. 2A). No significant difference was found in the expression of TRAAK in 17 term pregnant samples (38–40 weeks gestation) compared to 16 samples from non-pregnant myometrium (pre-menopause; age ≤43 yr) consistent with the results of gene expression for TRAAK (Fig. 1A). In a larger group of pregnant samples from both term (38–41 weeks gestation) and term laboring myometrium (38–41 weeks), no difference in TRAAK protein expression (Fig. 2B) was confirmed, consistent with gene expression data (Fig. 1C).

### TREK-1 Gene and Protein Expression

**Figure 3:**
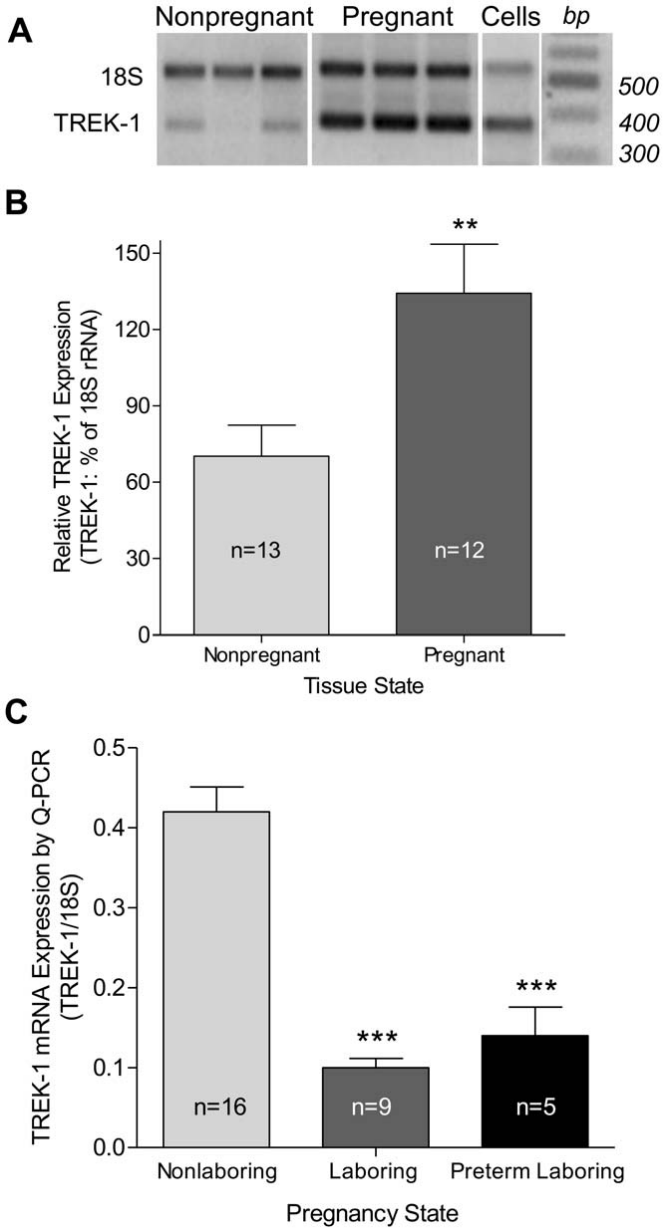
TREK-1 K2P channel expression in myometrium. (A) PCR studies were carried out on both non-pregnant and pregnant tissues and cells from term pregnancy myometrium and TREK-1 transcript confirmed by sequence analysis in each case. Individual lanes represent different patient samples and are shown to illustrate representative variation. (B) 18S ribosomal RNA was used to adjust TREK gene expression relative to control gene expression. (C) Expression of TREK in term and term laboring samples was determined by Q-PRC relative to the 18S ribosomal gene as an expression control (p<.001). Data are mean ± SEM; **  = p<0.01; ***  =  p<0.001 by unpaired T-test.

TREK-1 mRNA was expressed in both non-pregnant and pregnant human myometrial smooth muscle (Fig. 1A). Amplification of TREK-2 message from either non-pregnant or pregnant human myometrium yielded no evidence of expression consistent with the findings of Bai, et al. [20]. Ribosomal 18S RNA was used to normalize PCR reactions [15]. Expression of TREK-1 in isolated myometrial cells confirmed the origin of these channels as myocyte proteins (Fig 3A). TREK-1 mRNA expression varied significantly between non-pregnant and pregnant myometrium, demonstrating a ∼2 fold up-regulation during pregnancy toward term (70.2 *vs.* 134.2%; *p*<0.01; Fig. 3B) suggesting the possibility that TREK-1 plays a role during pregnancy. In order to determine the effect of labor at term, TREK-1 gene expression was examined in these patient samples by Q-PCR. The elevated expression seen in pregnant term samples when compared to laboring term samples revealed a dramatic decrease in gene expression consistent with the notion that TREK-1 contributes to myometrial quiescence at term (Fig. 3C). Because the absence of TREK-1 could contribute to preterm labor, we also determined TREK-1 expression in samples from women in labor at 28–33 weeks gestation. TREK-1 gene expression in preterm myometrium (Fig. 3C) was comparable to expression in laboring samples.

**Figure 4:**
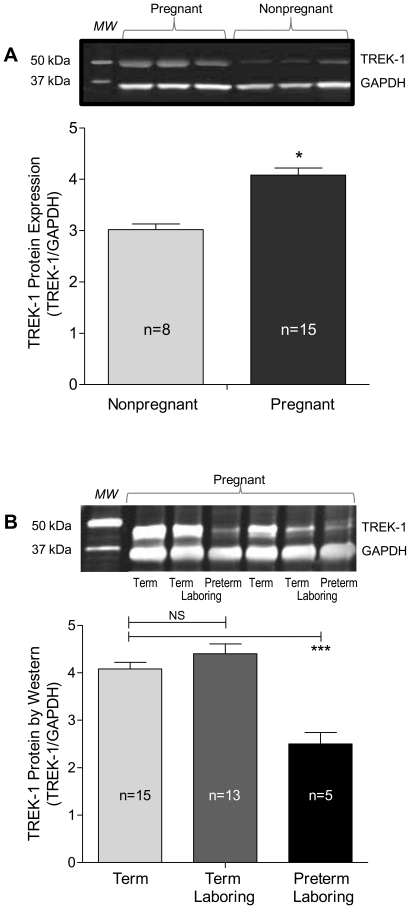
TREK-1 protein expression in myometrium. (A) Western blots were performed using a TREK-1-specific antibody (Santa Cruz) and stained using infrared fluorescence secondary. GAPDH was employed as an expression control using simultaneous imaging. Individual lanes represent different patient samples and are shown to illustrate representative variation. TREK-1 expression is significantly increased by pregnancy compared to non-pregnant samples and, (B) remains unchanged during labor but expression is lower in preterm samples. Data are mean ± SEM, *  =  p<0.05; ***  =  p<0.001 by unpaired T-test.

Translation of human myometrial TREK-1 gene into protein was confirmed in both pregnant and non-pregnant tissue samples (Fig. 4A) by Western blot quantified for both TREK-1 (48 kDa under reducing conditions) and GAPDH. TREK-1 protein expression showed a significant increase during pregnancy (26%, Fig. 4A) consistent with gene expression data (Fig. 3B). At the time of labor however, TREK protein expression measured by Western blot is still present at pre-laboring levels but is significantly lower in preterm samples (Fig. 4B).

### Inhibition of Stretch-Activated Channels Stimulates Myometrial Contractions

**Figure 5:**
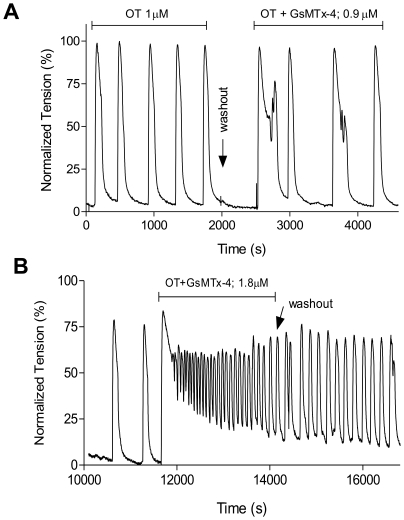
GsMTx-4 increases OT-induced contractions. Contractile bath experiments using the specific stretch-activated channel blocker, GsMTx-4 at doses of 0.9 to 1.8 µM, in the presence of OT were performed to observe the effect on non-laboring myometrium. OT-induced (1 µM) contractions were established, followed by a wash-out period. Tissue was then challenged with A) 0.9 µM GsMTx-4, causing increased duration of contractions, or (B) 1.8 µM GsMTx-4, causing attenuation of relaxation. Traces are representative of n = 4.

To verify the significance of stretch-activated channels to the physiological function of the myometrium, we explored the effects of inhibiting stretch-activated channels. In order to test the hypothesis that stretch-activated channels assist in the regulation of phasic contractions in myometrial smooth muscle, we utilized contractile bath studies. Increasing concentrations of GsMTx-4 (Grammostola spatulata mechanotoxin-4), a specific mechanosensitive channel blocker [35], amplified oxytocin (1 µM; OT) induced contractions in non-laboring pregnant myometrium. Addition of GsMTx-4 at 0.9 µM caused an increase in duration of contractions (Fig. 5A), while higher concentrations such as 1.8 µM toxin caused both an increase in duration and frequency of contractions (Fig. 5B). This effect was not seen on spontaneously contracting tissue strips (control, no OT treatment) and the degree of effect varied greatly from patient to patient but was consistently seen (data not shown).

**Figure 6:**
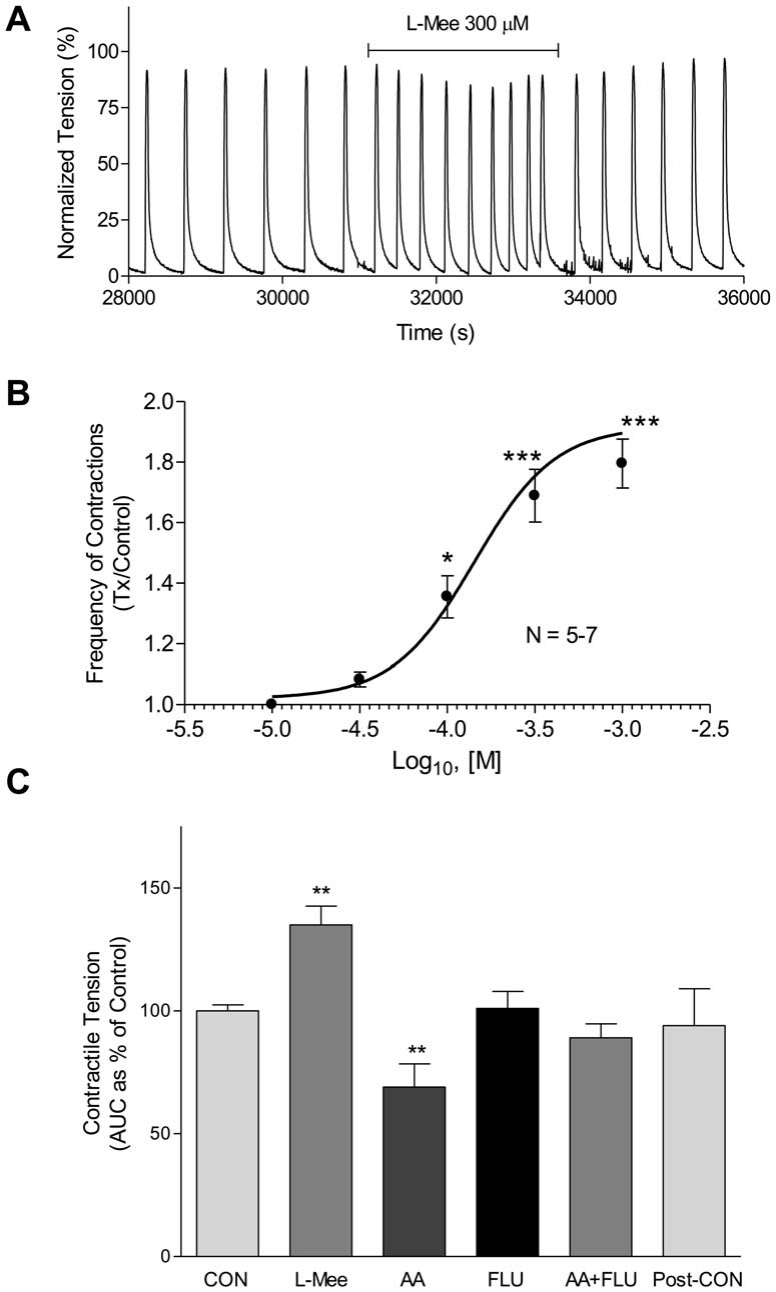
L-methionine ethyl ester increases frequency of contraction in a dose-dependent manner. L-Mee increased contractile frequency in non-laboring myometrial strips in a concentration-dependent manner (30 µM to 300 mM). Iberiotoxin (100 nM) was present to block Ca^2+^-activated currents and tissues established spontaneous contractions before drug addition. (A) 30 µM L-Mee applied to the bath caused a slight increase in frequency of contraction, with recovery after washout. (B) 100 µM L-Mee caused a significant increase in frequency of contraction that was pronounced at 300 µM L-Mee with recovery after washout; 1 mM L-Mee caused a marked increase in frequency of contraction with modest, variable reduction in peak amplitude that remained after washout (*not shown*). (C) In a similar set of tissues, arachidonic acid (AA) significantly depressed contractions, an effect blocked by fluphenazine (FLU; 100 µM), while addition of fluphenazine alone had no significant effect. The effect of L-Mee to increase contractions was not different when added in the presence of 100 µM AA (not shown). Tissues returned to control tension after washout. Data are mean ± SEM, **  =  p<0.05, n = 7.

Because GsMTx-4 affects all stretch-activated channels and thus in myometrium blocks both TREK-1 and TRAAK, we further tested the hypothesis that a TREK-1 more specific stretch-activated potassium channel inhibitor would diminish the ability of the myometrium to relax. It has been shown in bladder smooth muscle that methionine and its derivatives inhibit TREK channels, thereby increasing bladder excitability [17], [33]. We observed that increasing concentrations of L-methionine ethyl ester (L-Mee) augmented spontaneous contractions in non-laboring myometrium. Addition of L-Mee at 300 µM caused an increase in frequency of contractions (Fig. 6). We further normalized each tissue to itself under control conditions (100 nM iberiotoxin) and observed that L-Mee increased myometrial frequency of contraction in a dose dependent manner (Fig. 6B). L-Mee significantly inhibited myometrial relaxation (*i.e.* increased contractions) at concentrations ≥100 µM (*p*≤0.05) and reached maximal effect at ∼300 µM where contractile frequency was increased to ∼35% above control levels (Fig. 6C). At concentrations of L-Mee ≥1 mM we observed a reduction in peak contraction amplitude and a diminished ability for tissue recovery after washout of drug; tissues treated with 10 mM L-Mee failed to contract or relax (data not shown).

Fluphenazine and other antipsychotics have also been described as selective K2P blockers able to inhibit TREK-1 but not TRAAK channels [34]. We conducted contractile bath experiments in pregnant tissues using the K2P channel activator arachidonic acid (AA; 10 µM) and the putative TREK-1 inhibitor fluphenazine (FLU; 100 µM). OT (100 nM) stimulated contractions (ex. Fig. 6A) were blunted by the addition of AA compared to control consistent with TREK-1 channel activation (Fig. 6C). Addition of fluphenazine alone did not significantly alter contractility, while addition of FLU to oxytocin treated tissues in the presence of AA prevented the diminished contractility seen with AA alone (Fig. 6C). Tissues responded to OT stimulations in a fashion comparable to control after washout of AA and FLU (Fig. 6C. Post-Con). Concentrations of FLU greater than 300 µM suppressed OT-induced contractions and were irreversible during the course of the experiment suggesting non-specific effects (not shown).

## Discussion

While many factors are thought to modulate the contraction and relaxation of uterine smooth muscle, it is generally agreed that membrane ion channels are crucial to this process and are likely targets of many of the factors which regulate myometrial tone. Interest in stretch-activated channels in the myometrium stems from the knowledge that the uterus enlarges to accommodate a growing fetus during pregnancy. Evidence supports the general notion that potassium channels maintain the uterus in a quiescent state during gestation [36]. We further hypothesized that stretch activated potassium channels are differentially regulated and contribute substantially to the resting membrane potential in the pregnant myometrium, as well as serving to counteract contractile stimuli. We were therefore interested in elucidating the role and possible regulation of the stretch-activated channels, TREK and TRAAK. These channels and the factors that regulate them may provide a unique therapeutic target to regulate the contractility of the myometrium in cases such as preterm labor.

The stretch-activated channel TREK-1 was previously reported to only be minimally expressed in human myometrium [20], [28], [37], while the K2P channels TREK-2 and TRAAK were thought not to be expressed in this tissue at all [18], [20], [38]. Here we report that TREK-1 and TRAAK, but not TREK-2, transcripts and protein are expressed in human myometrium. Furthermore, TREK-1 but not TRAAK is substantially up-regulated in pregnant samples when compared to non-pregnant samples consistent with a functional role for TREK-1 channels in pregnancy. The stretch-activated channel toxin (GsM Tx-4) and the TREK-1 more selective channel inhibitors methionine ethyl ester and fluphenazine altered myometrial tension and contractile frequency consistent with the activity of TREK-1 currents in pregnancy myometrium. Increased TREK-1 channel expression as well as function in pregnant myometrium suggests that as pregnancy progresses and the stretch of the uterus increases, these K2P channels are activated to assist in the maintenance of relaxation. To the best of our knowledge, ours is the first work documenting the expression and action of TREK in the myometrium during human pregnancy and labor. This report of a regulated increase in both expression as well as channel function supports our hypothesis that TREK-1 channels are important in maintaining quiescence during pregnancy and that channel expression and or activation may be dysregulated in spontaneous pre-term labor.

This notion of differential expression of TREK-1 channels and their regulation of membrane excitability in normal physiology is supported by several reports in animal models. Differential regulation of TREK-1 has been shown in epicardial *vs.* endocardial myocytes in rat ventricle [39]. TREK-1 has further been observed to be developmentally regulated in rat ventricle with a suggested role in reducing cardiac excitability due to its hyperpolarizing effect [40]. Evidence also suggests the regulation of K2P channel expression and function in pathophysiological states. Elevated levels of TREK-1 mRNA and protein have been seen in hypertrophic myocardium [41]. TREK-1 and TRAAK have also been shown to be up-regulated in a rat model of experimental acute cerebral ischemia [42].

It is known that ischemia can activate phospholipase A_2_ and result in the accumulation of unsaturated fatty acids such as arachidonic acid. Accumulation of arachidonic acid would lead to activation of TREK, thereby causing an efflux of K^+^ and allow for membrane hyperpolarization to decrease cell excitability. This mechanism is hypothesized to provide a neuroprotective effect during cerebral ischemia and may parallel the mechanism by which we hypothesize the up-regulation of myometrial TREK-1 channels during gestation to help maintain the uterus in a relative state of quiescence.

Blockade of stretch-activated channels by GsMTx-4 in pregnant human myometrium increased contractions and subsequently attenuated normal relaxation mechanisms in non-laboring tissues. Stretch-activated K^+^ channels are likely to be significant contributors to the relaxed state in pregnancy because activation by arachidonic acid lowers myometrial tension in oxytocin-stimulated tissues, an effect blocked by fluphenazine while fluphenazine alone had no effect in our studies. Inhibition by L-Mee increases contractile frequency in non-laboring tissues consistent with removal of a relaxation influence on the muscle. Our findings are consistent with previous studies showing the inhibition of TREK-1 channels by methionine containing compounds or fluphenazine [17], [33], [34]. The mechanism of TREK-1 activation and inhibition in human myometrium is unknown and thus the failure of fluphenazine to alter contractions when added alone and yet block an effect of arachidonic acid are unexplained. These results may reflect distinctions in the manner of channel inhibition by these agents.

There is a clear dissociation between TREK-1 gene expression and protein expression in the samples tested (Fig. 3C vs. 4B). If our thesis is correct, this result may not reveal a distinction between gene regulation, reduced at the time of labor, and the presence of protein detectable in Western blots. While it is entirely likely that the time needed to see a fall in protein following changes in gene expression may not be provided for in laboring tissues, it is also possible that we do not detect labor-associated regulation of channel function such that continued presence of the protein is not inconsistent with labor. The decreased expression of both message and protein in preterm tissues however (Fig. 3C, 4B) is consistent with our hypothesis. Potassium channel internalization (a state of the channel not reflected by gene or protein expression), has been shown to be affected by post-translational modifications which can be differentially regulated depending on metabolic states [43]–[45]. In addition to regulation of K2P channels *via* expression levels and post-translational modulation, reports also support the presence of splice variants with differing channel activity. Alternative splicing of potassium channels has previously been shown to contribute to the diversity of channel specificity in myometrium [36], [46]–[48], as well as many other tissues [49]–[52]. More importantly, TREK-1 has been shown to be alternatively spliced in rat heart suggesting two different channel isoforms; one of lower (∼41 pS) and one of higher (∼132 pS) conductance, both stimulated at positive potentials [53]. This information, as well as a survey of available TREK-1 sequence variants on GenBank, leads us to hypothesize alternative splicing of TREK-1 in differing states of the uterus; a dysregulation or shift in predominant variant(s) and or assembly of channels as homo- or heterodimers in a regulated fashion by pregnancy may explain TREK-1 activation in pregnancy myometrium and alterations in this process may predispose certain women to spontaneous pre-term labor contributing to pre-term birth.

Difference in the expression of TREK-1 seen in pregnant versus non-pregnant myometrium are not thought to be the result of the age difference in patients in these two groups (Table 1). The fact that TRAAK expression was not different argues in favor of age not being a factor. However, age is significantly different between pre-gravid and gravid women but does not represent power sufficient to support a comparison of age difference in each pregnancy group versus pre-gravid women. We do not think it likely that age *per se*, influences our results.

In summary, our results provide strong evidence supporting the hypothesis that the K2P channel TREK-1 is functionally up-regulated in pregnancy, and differentially regulated during pregnancy to relax the uterus prior to labor. We hypothesize that TREK-1 is functionally dysregulated in spontaneous pre-term birth, contributing to the disruption of normal myometrial quiescence during pregnancy. This dysregulation may be the result of changes in channel expression levels, post-translational modulation and/or variant channel expression and dysfunctional channel assembly. TREK-1 channels and or their regulation offer promise as potential therapeutic targets in controlling pre-term uterine contractions.
